# Note on dose conversion for radon exposure

**DOI:** 10.1007/s00411-024-01077-0

**Published:** 2024-06-17

**Authors:** Thomas R. Beck

**Affiliations:** Federal Office for Radiation Protection, Berlin, Germany

**Keywords:** Radiation detriment, Radon, Effective dose, Dose conversion convention

## Abstract

The epidemiological approach to converting radon exposure to effective dose is examined. Based on the definition of the effective dose, the dose conversion is obtained from the equivalence of lung-specific detriment associated with low-LET radiation and with radon exposure. This approach most reliably estimates effective dose per radon exposure on the basis of epidemiological data and implicitly includes the radiation weighting factor required to calculate the effective dose from radon exposure using the dosimetric approach, applying biokinetic and dosimetric models. Consistency between the results of the epidemiological and dosimetric approaches is achieved by using a radiation weighting factor of about 10 for alpha particles instead of the current ICRP value of 20. In contrast, the epidemiological approach implemented in ICRP 65, and referred to as *dose conversion convention*, was based on direct comparison of total radiation detriment with lung detriment from radon exposure. With the revision of radiation detriments in ICRP 103, this approach can be judged to overestimate the effective dose per radon exposure by about a factor of two because the tissue weighting factor for lung differs from the value of relative detriment to which it relates.

## Introduction

Radiation detriment is used by the International Commission on Radiological Protection (ICRP) to represent the combination of the probability of occurrence of a harmful health effect and a judgement of the severity of that effect (ICRP 1991, 2007). Total radiation detriment values are presented as the sum of individual values for radiation-related cancers, derived principally on the basis of data from Life Span Study on Japanese atomic bomb survivors (LSS) with whole-body exposures mainly to gamma radiation. Gamma radiation is characterized by low-linear-energy-transfer (low-LET).

Conversion of radon exposure into effective dose is done by convention on the basis of the equivalence of detriments. Following this principle, ICRP 65 (ICRP 1993) established the *dose conversion convention* by “direct comparison of the detriment associated with a unit effective dose and a unit radon exposure”. For the detriment per unit effective dose, the total detriments of ICRP 60 (ICRP 1991) were used, but not the corresponding detriments for the lung. The detriment per unit radon exposure was derived from epidemiological studies on lung cancer risks of uranium miners and is expressed in units per Working Level Month (WLM). Since no detriment for radon exposure of the general public was available, the single detriment derived from miner studies was also applied to the dose conversion of the general public. It should be noted that ICRP 65 established the *dose conversion convention* by using the nominal fatality probability coefficient for radon and radon progeny-induced lung cancer as the detriment for radon. In later publications (e.g. ICRP 2010), the lifetime excess absolute risk (LEAR) is used as the nominal probability coefficient to reflect the lifetime detriment associated with exposure to radon and its progeny. The ICRP describes a risk quantity as “nominal” if it refers to the exposure of a defined population of females and males with a typical age distribution and is computed by averaging over age groups and both sexes (ICRP 2007).

The approach introduced in ICRP 65 to determine the epidemiologically based *dose conversion convention* is still used today, but with the total detriments updated in ICRP 103 (ICRP 2007): 4.2·10^–5^ per mSv for workers and 5.7·10^–5^ per mSv for the general public. The detriment for radon exposures is derived with more recent epidemiologic data from studies on miners (ICRP 2010; Marsh et al. 2021). In contrast to ICRP 65, there are now appropriate studies on residential radon.

ICRP has decided to recommend revised dose conversion coefficients for radon exposure using biokinetic and dosimetric models (dosimetric approach) (ICRP 2010). The same approach is thus applied for the intake of radon and its progeny as for other radionuclides. The use of the dosimetric approach is suggested by the good agreement between the conversion coefficients obtained from the *dose conversion convention* and the calculations with biokinetic-dosimetric models for underground miners (ICRP 2010, 2014, 2017).

This study examines the *dose conversion convention* for radon with respect to the definition of effective dose and aims to harmonize the approaches to calculating effective dose per radon exposure.

## Method

### Definition of effective dose

The effective dose,$E$, is defined as being the tissue-weighted sum of the equivalent doses in all specified tissues and organs of the body, given by the expression (ICRP 2007)

1 $$E = \sum\limits_{T} {\omega_{T} } \sum\limits_{R} {\omega_{R} D_{T,R} } = \sum\limits_{T} {\omega_{T} H_{T} } ,$$

where ${H}_{T}$ or ${\omega }_{R}{D}_{T,R}$ is the equivalent dose in a tissue or organ, $T$, and ${\omega }_{T}$ is the tissue weighting factor. ${D}_{T,R}$ represents the absorbed dose in the tissue or organ due to the radiation, $R$. The radiation weighting factor, ${\omega }_{R}$, is a dimensionless factor by which the organ or tissue absorbed dose is multiplied to reflect the higher biological effectiveness of high-LET radiations compared to low-LET radiations (different radiation qualities). It is assumed that the radiation weighting factor for a given radiation quality is the same for every organ or tissue.

The concept of effective dose is intended to establish a protective quantity that provides an measure of the radiation effects. It is applicable to different exposure scenarios in which the absorbed doses can be distributed homogeneously or inhomogeneously in the body and can result from radiations of different radiation quality. For this purpose, each organ or tissue is considered individually in Eq. (1). The absorbed doses received by the organ or tissue are each converted to the equivalent dose using the radiation weighting factor specified for the radiation quality under consideration and added together. Finally, the effective dose is the sum of the weighted equivalent doses of organs and tissues. The weights are determined by the tissue weighting factors.

Radon exposure results in an inhomogeneous dose distribution in the body with dose transfer predominantly to the lung tissue. The dosimetric approach not only takes into account the doses from radon progeny in the lungs, but also includes the doses received by all organs and tissues in the calculation. Even small additional contributions (< 5%) from the inhalation of radon gas are taken into account (ICRP 2017). However, epidemiological studies show no consistent evidence of an association between radon concentration and cancer, other than lung cancer (ICRP 2010). The detriment attributed to radon therefore arises from the inhalation of radon progeny, which leads to exposure of the lungs.

The delivered absorbed dose to the lung is caused by alpha particles released from the inhaled radon decay products. Alpha particle radiation is a high-LET radiation that has higher biological effectiveness compared to low-LET radiation at the same absorbed dose. This is expressed by the radiation weighting factor,${\omega }_{\alpha }$, for alpha particles. For the determination of the effective dose due to radon exposure, Eq. (1) simplifies to

2 $$E = \omega_{{{\text{Lung}}}} \omega_{\alpha } D_{{{\text{Lung,}}\,{\text{Radon}}}} = \omega_{{{\text{Lung}}}} H_{{{\text{Lung}}}} ,$$

where ${\omega }_{\text{Lung}}$ is the tissue weighting factor for the lung, and ${D}_{\text{Lung},\text{Radon}}$ the absorbed dose imposed on lung tissue by alpha particles from radon decay products. In particular, Eq. (2) is used in biokinetic and dosimetric models to calculate the effective dose resulting from exposure to the lung.

### Equivalence of detriments from low-LET radiation and radon exposure

Application of Eq. (2), requires determination of the lung equivalent dose from low-LET radiation, ${H}_{\text{Lung}}$, which produces the same radiation effects as the corresponding absorbed dose from radon and radon progeny, $H_{{{\text{Lung}}}} = \omega_{\alpha } D_{{{\text{Lung}},\,{\text{Radon}}}}$. For this purpose, the convention on the equivalence of lung detriment provided by ICRP and associated with exposure to low-LET radiation, ${\mathfrak{D}}_{{{\text{Lung}}}}$, and lung detriment from radon exposure, ${\mathfrak{D}}_{{{\text{Lung,}}\,{\text{Radon}}}}$, shall be used,

3 $${\mathfrak{D}}_{{{\text{Lung}}}} = {\mathfrak{D}}_{{{\text{Lung,}}\,{\text{Radon}}}} .$$

This convention differs from the *dose conversion convention* used in ICRP 65 and thereafter, where the total radiation detriment given in ICRP 60 or later in ICRP 103 is compared with the detriment from radon exposure. Radiation detriments are determined from the risk coefficients, taking into account the severity of the disease in the form of lethality and years of life lost. They correlate with the absorbed dose transferred to the organ by the radiation (ICRP 2007; Beck 2022).

Assuming a linear relationship between respective dose and radiation detriment, Eq. (3) can be extended,

4 $$\left( {\frac{{\mathfrak{D}}}{D}} \right)_{{{\text{Lung}}}} D_{{{\text{Lung}}}} = \left( {\frac{{\mathfrak{D}}}{D}} \right)_{{{\text{Lung}},\,{\text{Radon}}}} D_{{{\text{Lung}},\,{\text{Radon}}}} .$$

${\left(\mathfrak{D}/D\right)}_{\text{Lung}}$ is the coefficient of the lung detriment per absorbed dose from low-LET radiation. Since the radiation weighting factor for low-LET radiation (e.g. gamma radiation) is equal to 1 by convention, the absorbed dose is identical to the equivalent dose, ${D}_{\text{Lung}}={H}_{\text{Lung}}$, and ${\left(\mathfrak{D}/D\right)}_{\text{Lung}}$ can be replaced by ${\left(\mathfrak{D}/H\right)}_{\text{Lung}}$ without changing the value of Eq. (4).

From Eq. (4) and using the tissue weighting factor, the effective dose follows as

5 $${\text{E}} = \omega_{{{\text{Lung}}}} H_{{{\text{Lung}}}} = \omega_{{{\text{Lung}}}} \frac{{\left( {{\mathfrak{D}}/D} \right)_{{{\text{Lung,}}\,{\text{Radon}}}} }}{{\left( {{\mathfrak{D}}/H} \right)_{{{\text{Lung}}}} }}D_{{{\text{Lung,}}\,{\text{Radon}}}} .$$

Substituting the absorbed dose due to inhalation of radon progeny by radon exposure, ${D}_{\text{Lung},\text{Radon}}=f\cdot {\mathcal{X}}_{\text{Radon}}$, where ${\mathcal{X}}_{\text{Radon}}$ represents the radon exposure and $f$ is a constant factor indicating the absorbed dose to the lung per radon exposure derived from biokinetic and dosimetric models, it follows

6 $$E = \omega_{{{\text{Lung}}}} \cdot\frac{{\left( {{\mathfrak{D}}/{\mathcal{X}}} \right)_{{{\text{Lung,}}\;{\text{Radon}}}} }}{{\left( {{\mathfrak{D}}/H} \right)_{{{\text{Lung}}}} }}{\mathcal{X}}_{{{\text{Radon}}}} .$$

In addition, the coefficient of the lung detriment per equivalent dose, ${\left(\mathfrak{D}/H\right)}_{\text{Lung}}$ can be converted into the total detriment. For this purpose, the relative detriment, ${\mathfrak{d}}_{\text{rel},\text{T}}$, is used given as relationship between the organ- or tissue-specific detriment (index: T) and the total detriment (index: tot),

7 $${\mathfrak{d}}_{{{\text{rel}},{\text{T}}}} = \frac{{\left( {{\mathfrak{D}}/H} \right)_{{\text{T}}} }}{{\left( {{\mathfrak{D}}/H} \right)_{{{\text{tot}}}} }} .$$

Applying Eq. (7) for the lung ($\text{T}=\text{Lung})$ and inserting it into Eq. (6), the lung detriment for low-LET radiation is replaced by the total detriment and the effective dose is obtained from the radon exposure by

8 $${\text{E}} = \frac{{\omega_{{{\text{Lung}}}} }}{{{\mathfrak{d}}_{{{\text{rel,}}\,{\text{Lung}}}} }}\cdot\frac{{\left( {{\mathfrak{D}}/{\mathcal{X}}} \right)_{{{\text{Lung,}}\,{\text{Radon}}}} }}{{\left( {{\mathfrak{D}}/H} \right)_{{{\text{tot}}}} }}{\mathcal{X}}_{{{\text{Radon}}}}$$

## Results

The coefficient for the conversion of radon exposure into the effective dose follows from Eq. (8) after simple rearrangement,

9 $$\frac{E}{{{\mathcal{X}}_{{{\text{Radon}}}} }} = \frac{{\omega_{{{\text{Lung}}}} }}{{{\mathfrak{d}}_{{{\text{rel,}}\,{\text{Lung}}}} }} \cdot \frac{{\left( {{\mathfrak{D}}/{\mathcal{X}}} \right)_{{{\text{Lung,}}\,{\text{Radon}}}} }}{{{\mathfrak{D}}/H_{{{\text{tot}}}} }}$$

The equation shows that the conversion of radon exposure to dose is not only a “direct comparison of the detriment associated with a unit effective dose and a unit radon exposure”, as introduced by ICRP 65. An additional factor indicating the relationship between tissue weighting factor and relative lung detriment must be considered. This factor is a necessary correction to account for the fact that the contribution of the lung dose to the effective dose given by ${\omega }_{\text{Lung}}$ is not the same as its contribution to the total detriment given by ${\mathfrak{d}}_{\text{rel},\text{Lung}}$. Equation (9) demonstrates that the ICRP epidemiological *dose conversion convention* only leads to an effective dose that is consistent with its definition if ${\omega }_{\text{Lung}}{=\mathfrak{d}}_{\text{rel},\text{Lung}}$.

It should be noted that dose conversion can alternatively be determined from the ratio of the lung-specific detriments according to Eq. (6).

From the comparison of Eq. (5) with Eq. (2), the ratio of the corresponding detriment coefficients for the lung is identified as the radiation weighting factor for alpha particles caused by radon and radon progeny,

10 $$\omega_{{\upalpha }} = \frac{{\left( {{\mathfrak{D}}/D} \right)_{{{\text{Lung}},\,{\text{Radon}}}} }}{{\left( {{\mathfrak{D}}/H} \right)_{{{\text{Lung}}}} }}$$

Lung detriments from low-LET radiation and radon exposure determine the radiation weighting factor, allowing the results of epidemiological studies on health effects to be taken into account in dose calculation. Equation (10) has already been used elsewhere for this purpose (Harrison and Muirhead 2003; Marsh et al. 2014; ICRP 2021).

## Discussion

### Relationship between tissue weighting factor and relative lung detriment

ICRP has defined a set of tissue weighting factors, including the lung factor, ${\omega }_{\text{Lung}}$. The factors are averages over many individuals of both sexes and are intended to apply for the whole and the working age population. It could be proposed that they closely follow the respective values of relative detriment. However, ICRP has made simplifications and roundings in order to obtain a single set of only four tissue weighting factors for use in radiation protection. The tissue weighting factor assigned to a specific organ may therefore differ from the corresponding relative detriment (ICRP 2007). The weighting factors recommended by the ICRP for calculating the effective dose are often also prescribed for use in national legislation (EU Council Directive 2013).

Table 1 compiles the tissue weighting factors and the relative lung detriments for the whole population and the working age population published by ICRP. In ICRP 60, radiation detriments for the public and for workers were derived based on mortality data from five selected populations and the risk models from the LSS. Considering the values of ICRP 60, the tissue weighting factor agreed quite well with the relative lung detriment and therefore the condition ${\omega }_{\text{Lung}}{=\mathfrak{d}}_{\text{rel},\text{Lung}}$ is approximately satisfied. Because of the good agreement between the relative detriment and the tissue weighting factor for the lung with a ratio of about 1, the *dose conversion convention* proposed by ICRP 65 allowed easier conversion of radon exposure to effective dose by direct comparison of the detriment from radon exposure to the total radiation detriment.

**Table 1:** Comparison of values for tissue weighting factor as well as coefficients for total radiation detriments and lung-specific radiation detriments according to ICRP 60 and ICRP 103

		Total radiation detriment ${\left(\mathfrak{D}/H\right)}_{\text{tot}}$ 10^–5^ mSv^−1^	Lung-specific values			
			Lung detriment ${\left(\mathfrak{D}/H\right)}_{\text{Lung}}$ 10^–5^ mSv^−1^	Relative detriment ${\mathfrak{d}}_{\text{rel},\text{Lung}}$	Tissue weighting factor ${\omega }_{\text{Lung}}$	Ratio $\frac{{\omega }_{\text{Lung}}}{{\mathfrak{d}}_{\text{rel},\text{Lung}}}$
Whole population	ICRP 60	7.3^a^	0.80^a^	0.110	0.12	1.09
	ICRP 103	5.74^b^	0.903^b^	0.157^b^	0.12	0.76
Working age population	ICRP 60	5.6^a^	0.64^a^	0.114	0.12	1.05
	ICRP 103	4.22^b^	1.207^b^	0.286^b^	0.12	0.42

^a^ICRP 60, Table 4

^b^ICRP 103, Table A.4.1

**Fig. 1:**
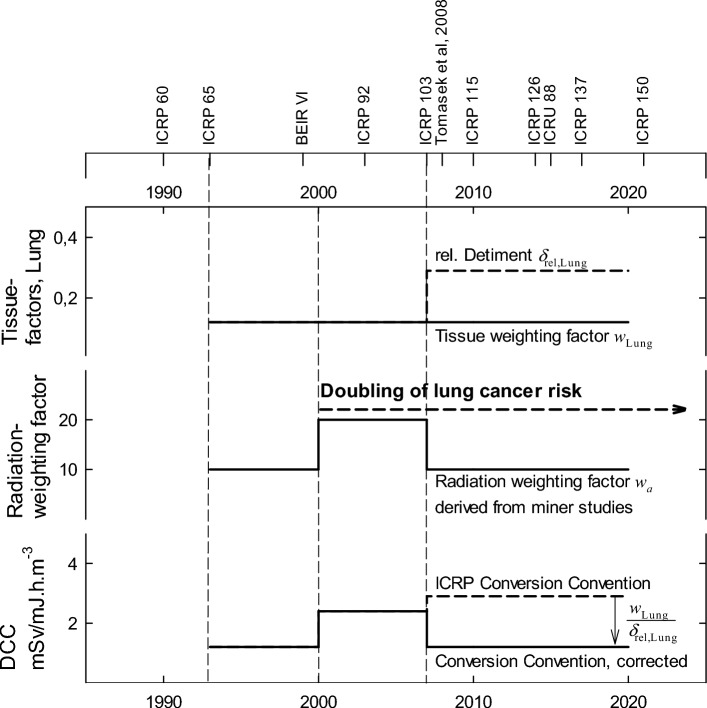
Chronology of publications and changes in the factors since 1990 (factors given for working age population, *DCC* dose conversion coefficient), figure is for visualization purposes only, values are approximated

As a result of scientific progress, the methodology, data, and risk models for calculating detriments have changed. Figure 1 summarizes the chronology of relevant publications and the changes in the factors since 1990. ICRP 103 estimated the risks for radiation-induced cancer incidence and the resulting detriments in Asian and Euro-American representative populations. While the tissue weighting factor has not changed, Table 1 and Fig. 1 show that the calculations performed in ICRP 103 significantly increased the relative lung detriment. Consequently, the tissue weighting factor no longer coincided with the relative lung detriment and the ratio ${\omega }_{\text{Lung}}{/\mathfrak{d}}_{\text{rel},\text{Lung}}$ fell well below 1.

### Relationship between the lung detriments from low-LET radiation and radon exposure

According to Eq. (10), a radiation weighting factor of about 10 was implicit in the dose conversion of ICRP 65 (Harrison and Muirhead 2003). Subsequent epidemiological studies on uranium miners revealed a doubling of the health risk from radon compared to the results of the epidemiological study used in ICRP 65 (NRC 1999; Tomasek et al. 2008). This became evident from around the year 2000. ICRP 115 later adopted the detriment-adjusted nominal risk coefficient of 5∙10^–4^ per WLM, which almost doubled the value of 2.83·10^–4^ per WLM from ICRP 65. It increased the numerator of Eq. (10), resulting in an increase in the implied radiation weighting factor and an approximate doubling of the dose conversion coefficient after the year 2000.

With the publication of ICRP 103, the new lung detriment for the working age population was about twice that of ICRP 60 (Table 1). This increased the denominator of Eq. (10), which eventually compensated for the previous doubling of the nominator. As a result, the value of the radiation weighting factor according to Eq. (10) was reduced to approximately the same value already implied in ICRP 65 (Marsh et al. 2014; ICRU 2015). In agreement to Eq. (6) or Eq. (9), this should also be accompanied by a corresponding reduction in the dose conversion coefficient. However, retaining the *dose conversion convention* from ICRP 65 of directly comparing the total radiation detriment to the detriment from radon exposure ignored these changes and thus can be seen to overestimate the dose conversion coefficient by a factor of about two.

### Implications for dose conversion associated with radon exposure

The new epidemiological evidence on radon confirms the radiation weighting factor of about 10, which was already implicit in the ICRP 65 dose conversion for radon exposure. This factor is half the factor of 20 recommended by the ICRP for alpha particles and currently used in the dosimetric approach. The radiation weighting factor based on epidemiological studies of radon is more meaningful for use with radon exposures than the non-specific ICRP factor. If the lower factor were included in the dosimetric approach, the dose conversion would be half the value recommended for workplaces in ICRP 137 (ICRP 2017).

Consistency between the dose conversion for radon exposures in mines derived from the epidemiological approach and the dose conversion derived from the dosimetric approach would still be preserved if the difference between the tissue weighting factor and the relative lung detriment according to Eq. (9) were taken into account.

## Conclusion

This study examined the epidemiological approach for converting radon exposure to effective dose. It was shown that dose conversion based on the equivalence of lung-specific detriment from low-LET radiation and from radon exposure allows determination of the effective dose according to its definition. The epidemiological studies on radon provide direct evidence on the relative effectiveness of alpha particles in causing lung cancer and currently tend to yield a radiation weighting factor of about 10, which is half of the factor recommended by the ICRP for alpha radiation. The use of a radiation weighting factor of about 10 in the dosimetric approach would maintain consistency between the dose conversions derived from the epidemiologic and dosimetric approaches, thereby approximately confirming the dose conversion coefficient already derived in ICRP 65.

The epidemiological approach introduced in ICRP 65 and referred to as *dose conversion convention* is based on the direct comparison of total radiation detriment with lung detriment from radon exposure. The continued application of the *dose conversion convention* using current data justifies the adoption of the dosimetric approach with a radiation weighting factor of 20 for the determination of the dose coefficients for radon. However, the convention ignores the existing difference between the tissue weighting factor and the relative lung detriment, which became significant after the revision of the calculation method for the radiation detriment in ICRP 103. Therefore, the epidemiological approach based on the ICRP 65 *dose conversion convention* can be seen to overestimate the effective dose due to radon exposure. Consequently, it appears unsound to use the similarity between this overestimate and dose coefficients obtained by the dosimetric approach in support of the use of these coefficients. Consequently, the *dose conversion convention* cannot be used to justify the dose coefficients for radon exposure derived from the dosimetric approach.

The radiation weighting factor for alpha particles has been the subject of scientific debate for some time. A radiation weighting factor of 10 appears to be more realistic for a mixed population exposed to radon and its progeny (Harrison and Muirhead 2003; Hofmann et al. 2004; Marsh et al. 2014; ICRU 2015). For Euro-American males exposed in mines, a factor of about 14 has recently been determined (ICRP 2021). ICRP 92 already considered the radiation weighting factor of 20 for alpha particles as a guideline value and recommended that a more meaningful radiation weighting factor must be derived for certain situations such as radon and its progeny. This can be done on the basis of epidemiological information (ICRP 2003). The present study explicitly supports the application of this concept in order to improve the consistency between the effective dose determined by the dosimetric approach and the epidemiological risk estimates for radon exposure.

No datasets were generated or analysed during the current study.
